# The Bxb1 recombination system demonstrates heritable transmission of site-specific excision in *Arabidopsis*

**DOI:** 10.1186/1472-6750-12-9

**Published:** 2012-03-21

**Authors:** James G Thomson, Ronald Chan, Jamison Smith, Roger Thilmony, Yuan-Yeu Yau, YueJu Wang, David W Ow

**Affiliations:** 1 Crop Improvement and Utilization Research Unit, Western Regional Research Center, USDA-ARS, 800 Buchanan Street, Albany, CA 94710, USA; 2 Plant Gene Expression Center and UC Berkeley, 800 Buchanan Street, Albany, CA 94710, USA

## Abstract

**Background:**

The mycobacteriophage large serine recombinase Bxb1 catalyzes site-specific recombination between its corresponding *attP *and *attB *recognition sites. Previously, we and others have shown that Bxb1 has catalytic activity in various eukaryotic species including *Nicotiana tabacum*, *Schizosaccharomyces pombe*, insects and mammalian cells.

**Results:**

In this work, the *Bxb1 *recombinase gene was transformed and constitutively expressed in *Arabidopsis thaliana *plants harboring a chromosomally integrated *attP *and *attB-*flanked target sequence. The Bxb1 recombinase successfully excised the target sequence in a conservative manner and the resulting recombination event was heritably transmitted to subsequent generations in the absence of the recombinase transgene. In addition, we also show that Bxb1 recombinase expressing plants can be manually crossed with *att*-flanked target transgenic plants to generate excised progeny.

**Conclusion:**

The Bxb1 large serine recombinase performs site-specific recombination in *Arabidopsis thaliana *germinal tissue, producing stable lines free of unwanted DNA. The precise site-specific deletion produced by Bxb1 *in planta *demonstrates that this enzyme can be a useful tool for the genetic engineering of plants without selectable marker transgenes or other undesirable exogenous sequences.

## Background

With the demonstration that foreign DNA can be stably introduced into plant cells in the 1980's, the generation of transgenic plants has become a commercial industry and its use in agriculture has continually grown with production surpassing 134 million Ha in 25 countries [1]. The most commonly enhanced traits being herbicide tolerance, pest resistance, or both traits stacked together [2]. The advancement of plant genetic engineering has led to more sophisticated techniques and strategies being utilized for value added crop production. For many years it has been known that the structure of a transgene locus can have a major influence on the level and stability of transgene expression. Therefore researchers have studied methods for precise DNA integration with particularly interest in how to stack transgenes 'cleanly' to prevent interaction with the plant's genome defense and silencing systems. The idea of 'clean' integration and or stacking benefits commercialization for it addresses concerns by the public and regulatory authorities [3]. Therefore as the practices of transgenic technology are coming under greater scrutiny, research focusing on eliminating marker genes and vector sequences, and controlling the integration of transgenes with regard to copy number, orientation, and rearrangements has been pursued. Site-specific recombinase technology is a prominent tool for improving the precision by which crops are genetically engineered. In the last several years, recombinases have been shown to eliminate unneeded DNA, resolve multimers and perform site-specific targeted integration in a variety of species.

The novel site-specific recombinase Bxb1, isolated from mycobacteriophage Bxb1, belongs to the large serine recombinase sub-family [4]. Large serine recombinases act on two unique sequences, known as the recognition sites *attP *and *attB*, to yield the product sites known as *attL *and *attR *[4]. Depending on the relative orientation of the *attP *and *attB *sites, the reaction can result in excision, inversion or integration of sequences between the recognition sites, and is not reversible unless an additional protein, an excisionase, is present. Several recombinase systems of this type including Bxb1, TP901-1, U153 [5-7] and phiC31 [8], have been shown to function in eukaryotic cells. The Bxb1 recombinase is a 500 amino acid protein that binds minimal recognition sites *attP *and *attB *that are 39 bp and 34 bp, respectively and enzymatically executes uni-directional site-specific recombination [9]. *In vitro *studies on the Bxb1 system have shown that it can catalyze site-specific recombination in the absence of other proteins or high-energy cofactors [10]. The first plant study on the Bxb1-*att *system demonstrating its functionality was conducted in tobacco protoplasts [7]. The Bxb1 system has also been shown to function in insect and mammalian tissue culture cells [11-13]. The uni-directional mode of action of the Bxb1-*att *system is an impotant attribute that differentiates its utility as a tool for genomic manipulation from other recombinase systems. For example, recombinases from the the small tyrosine family which have been shown to function in plants (i.e. Cre, FLP, R) all naturally perform bidirectional or fully reversible recombination reactions.

In previous studies, we identified a number of prokaryotic site-specific recombination systems that function in the eukaryote *Schizosaccharomyces pombe *[8]. Among those, the Bxb1 uni-directional recombinase was highly efficient. The system has been successfully shown capable of recombinase mediated excision, inversion and integration reactions. While published evidence has demonstrated the Bxb1 system can mediate integration events in tobacco protoplasts [7], to our knowledge, heritable excision *in planta *has not been yet reported.

In this research, we examined the ability of the Bxb1-*att *recombination system to mediate genomic site-specific excision from within the *Arabidopsis *germline. Plants transgenic for an *attP *and *attB *flanked target sequence were generated and subsequently retransformed with a second construct carrying the *Bxb1 *transgene. The ability of the Bxb1 recombinase to perform excision of the target sequence from four independent plant lines (i.e. four unique genomic locations) and generate stably excised progeny plants that carry only the recombined target DNA of interest was assessed. Here, we report on the efficiency by which the excision event was heritably transmitted to subsequent generations in the absence of the recombinase gene.

## Results

### Experimental design

**Figure 1:**
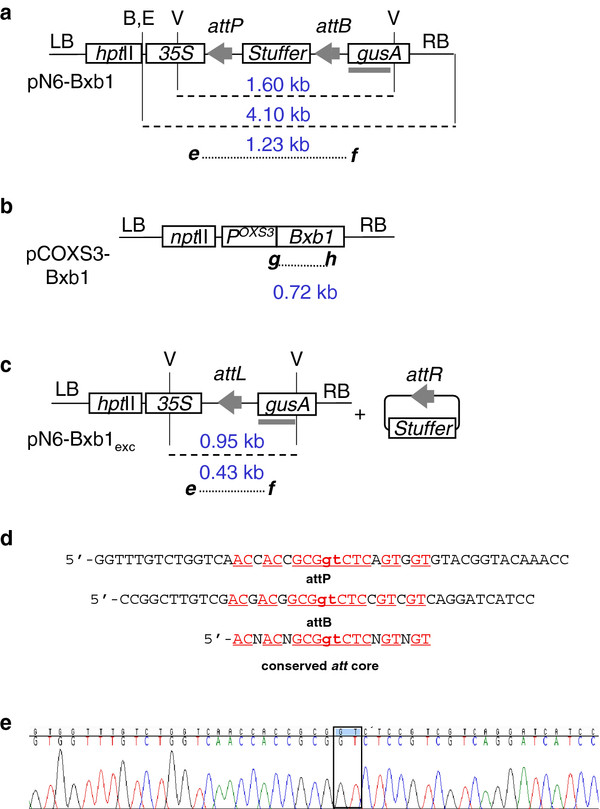
**T-DNA structures (not to scale) from a) pN6-Bxb1; b) pCOXS3-Bxb1; and c) predicted single copy T-DNA structures after excision of stuffer by Bxb1-*att *recombination**. PCR primers shown as *e*, *f*, *g, h*; *att *sites as grey arrowheads; hybridization probes as grey rectangles. Abbreviations: B, *Bam*HI; E, *Eco*RI; V, *Eco*RV, RB, T-DNA right border; LB, T-DNA left border, *P^OXS3 ^Arabidopsis OXS3 *promoter. Length in kb of PCR products (dotted lines) and DNA fragments (dashed lines). d) Sequence of the 51 bp *attP *and 42 bp *attB *Bxb1 recognition sites, where the minimal required sequence is underlined and the 2 nucleotide '**gt**' core region of crossover is in bold. e) sequence of a PCR product detecting a conservative site-specific excision event. Not shown are gene terminators and promoters for *hpt*II (hygromycin phosphotransferase II) and *npt*II (neomycin phosphotransferase II) and *gusA *(β-glucuronidase).

To test for site-specific recombination, we initially sought to use a gain-of-function strategy whereby excision of a transgene would lead to promoter fusion with a previously distal marker [14]. Hence, pN6-Bxb1 was configured with a CaMV 35*S *promoter (*35S*) proximal to a 760 bp stuffer region followed by a distal *gusA *coding region (Figure 1a). The stuffer region is flanked by directly oriented *attP *and *attB *recognition sites of the Bxb1 recombinase. The expectation was that prior to site-specific recombination, *35S *would not drive expression of *gusA *due the presence of the stuffer region. After recombination, the stuffer would be removed and activate expression of the *gusA *reporter gene (Figure 1c). In this strategy, we first introduced the recombination target (pN6-Bxb1) into the *Arabidopsis *genome via *Agrobacterium *transformation. These target lines, or 'TA' lines, were then transformed with the second construct, pCOXS3-Bxb1 (Figure 1b) that expresses the recombinase gene to produce the 'TR' lines. Upon site-specific excision of the recognition site-flanked DNA, the TR_1 _plants were back crossed to wild type plants and the BC_1 _progeny screened for segregants that retain the excision event but lack the recombinase gene. Lines positive for excision but negative for the recombinase gene were self-fertilized. To remove possible *de novo *recombinase expression contamination to the BC_1 _results the 'selfed' progeny (S_1_) lines were analyzed as a final confirmation for germinal transmission. A similar strategy was employed in previous recombinase research on phiC31 [6].

### Target lines for Bxb1 recombination

The target construct pN6-Bxb1 was introduced into *Arabidopsis*, and 44 hygromycin resistant lines were confirmed by PCR detection of a 1.23 kb product that spans the recognition site-flanked stuffer region (data not shown). Of those, 23 pN6-Bxb1 lines were propagated to the TA_2 _generation and examined by Southern blot for single copy T-DNA integration. *Eco*RI or *Bam*HI each cuts once within the target T-DNA (Figure 1a). Hybridization with a *gusA *probe of *Eco*RI or *Bam*HI cleaved genomic DNA should reveal a band size > 4.10 kb, the length of the cleaved T-DNA. A hybridizing band < 4.10 kb would indicate integration of a truncated T-DNA. From this analysis, 4 of the 23 pN6-Bxb1 plants were determined to contain a single copy of a likely complete T-DNA (data not shown) and designated TA_2_-Bxb1.9, 12, 28 and 35. The 1.23 kb PCR product from each of these lines was sequenced to confirm the presence of intact *attP *and *attB *sites (Figure 1d).

### Arabidopsis OXS3 promoter for expression of Bxb1

As previous research has demonstrated successful germline tissue expression of other recombinase genes [15], we again chose the 1.5 kb promoter fragment of the *Arabidopsis Oxidative Stress 3*gene (*OXS3*, At5g56550) [16] for *Bxb1 *transgene expression and generated the plasmid construct pCOXS3-Bxb1 (Figure 1b).

### Secondary transformation of TA target lines

**Figure 2:**
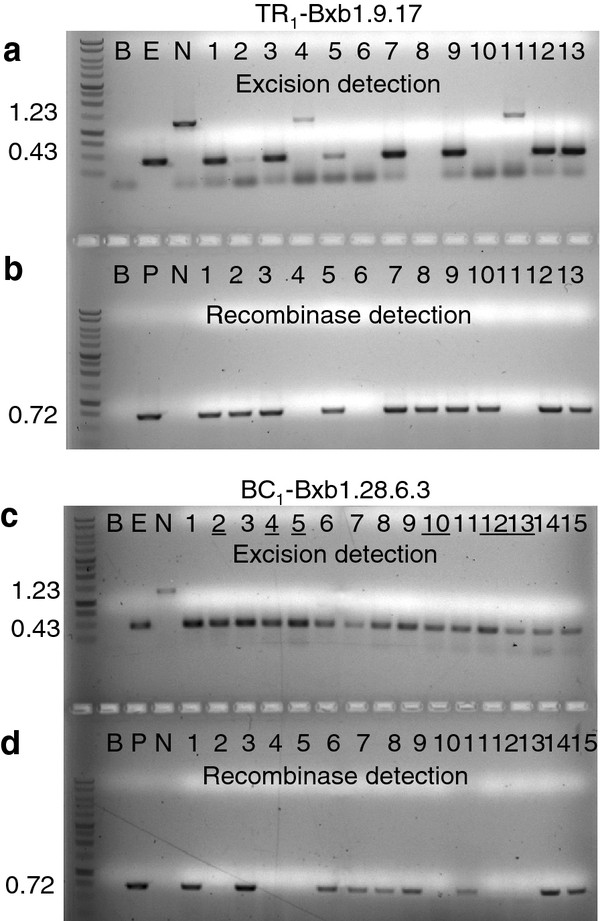
**PCR analysis for detection of site-specific recombination and the recombinase transgene in the retransformation (TR_1_) and back cross (BC_1_) generations**. PCR reactions (**a, c**) with primers ***e ***and ***f ***(Figure 1) or (**b, d**) with primers ***g ***and ***h ***(Figure 1) on representative plant DNAs. **a, b**) retransformed TR_1_-Bxb1.9.17 lines. **c, d**) Back crossed line BC_1_-Bxb1.28.6.3. Control lanes are B (blank, no DNA); E (excision, pN6-Bxb1_exc _DNA); N (no excision, pN6-Bxb1); P (pCOXS3-Bxb1). **c**) Lane numbers underlined indicate presence of excision event in the absence for the *Bxb1 *gene.

The TA_3 _generation of Bxb1.9, 12, 28 & 35 plant lines were transformed with *Agrobacterium *harboring the pCOXS3-Bxb1 vector producing the TR_1 _plant lines. Kanamycin resistant transformants that exhibited a wild type appearance and growth rate were identified and grown in the greenhouse. Three-week old TR_1 _transformants were tested for the presence of the *Bxb1 *gene. PCR amplification by primers ***g ***and ***h ***(Figure 1b) showed that a majority of the plants harbor the recombinase gene (Figure 2b). The groups of plants that harbor the *Bxb1 *gene were designated TR_1_-Bxb1.9, 12, 28 and 35 (Table 1).

**Table 1:** PCR analysis of TR_1 _plants

TA Parent line	Plants tested	Positive for recombinase gene *^a ^*and target locus *^b^*	**Positive for excision **^**c**^	Positive for **excision and negative for unexcised product**^***d***^
Bxb1.9	48	28	28	17

Bxb1.12	78	31	28	26

Bxb1.28	18	18	18	16

Bxb1.35	44	28	26	13

*^a ^*Primers ***g ***and ***h ***yielded the 0.72 kb *Bxb1 *fragment

*^b ^*Primers ***e ***and ***f ***yielded the 1.23 and/or 0.43 kb fragment

*^c ^*Primers ***e ***and ***f ***yielded the 0.43 kb excision fragment

*^d ^*Primers ***e ***and ***f ***failed to detect a 1.23 kb target fragment

The TR_1_-Bxb1 lines were examined using histochemical staining to detect *gusA *encoded reporter gene activity. The TR_1_-Bxb1 lines, however, exhibited variable levels of β-glucuronidase activity. One interpretation of this reduced activity is that the lines were undergoing low levels of Bxb1-mediated excision, but PCR analysis of lines where GUS activity was weak or undetectable were positive for excision of the target DNA. Given that the screening for GUS activity was not a reliable indicator of Bxb1 site-specific recombination, we subsequently utilized PCR to screen for site-specific excision.

With the 48 TR_1_-Bxb1.9, 78 TR_1_-Bxb1.12, 18 TR_1_-Bxb1.28 and 44 TR_1_-Bxb1.35 individuals, PCR with primers ***e ***and ***f ***(Figure 1c) detected a 0.43 kb product expected for site-specific excision (Figure 2, Table 1). However, the 1.23 kb product representing the parental configuration was also detected in some individuals, which indicates the presence of unexcised target DNA. As each individual harbors an independent COXS3-Bxb1 T-DNA integration at a different genomic location, with perhaps a different copy number or structural arrangements, the incomplete excision in some individuals was likely due to variability in recombinase gene expression. Based on the genomic PCR results, 35-89% of the *Bxb1 *positive TR_1 _plants generated only the 0.43 kb excision PCR product (Table 1).

### Segregation of the Bxb1 gene

To determine if the genomic excision event occurred in the germline tissue, we examined whether the excised target was heritably transmitted to the progeny lacking the *Bxb1 *gene. This analysis also determines whether or not the excision reaction was generated *de novo *in each generation. Individuals were chosen from each of the TR_1_-Bxb1.9, TR_1_-Bxb1.12 TR_1_-Bxb1.28 and TR_1_-Bxb1.35 families (Table 2) to pollinate wild type recipients. The backcross progenies (BC_1_) were grown without selection and then screened by PCR for the target locus (primers ***e ***and ***f***) and the recombinase gene (primers ***g ***and ***h***), which reveals whether excision occurred (0.43 kb band) or not (1.23 kb band) and if *Bxb1 *was present or absent. With the TR_1_-Bxb1.9, TR_1_-Bxb1.12, TR_1_-Bxb1.28 and TR_1_-Bxb1.35, 75% (229 of 306), 65% (70 of 107), 95% (156 of 165) and 73% (222 of 306) of the BC_1 _plants harbored the target DNA, respectively (Table 2).

**Table 2:** PCR analysis of BC_1 _and S_1 _plants

TR_1_-Parent line	Plants tested	**Positive for target locus **^***a***^	**Positive for excision **^***b***^	**Positive for excision and negative for recombinase gene **^***c***^	**Positive for recombinase gene and negative for target locus **^***d***^
Bxb1.9.7	48	36	20	0	1

Bxb1.9.8	18	14	14	5	0

Bxb1.9.11	6	6	6	0	0

Bxb1.9.17	40	32	32	15	1

Bxb1.9.23	51	41	36	0	9

Bxb1.9.26	49	46	22	0	0

Bxb1.9.29	18	16	12	0	1

Bxb1.9.31	44	10	9	0	24

Bxb1.9.44	32	28	26	17	0

Bxb1.12.52	27	14	8	0	6

Bxb1.12.61	9	6	6	1	2

Bxb1.12.69	8	2	2	0	3

Bxb1.12.72	9	4	1	0	3

Bxb1.12.73	9	3	3	1	6

Bxb1.12.74	9	9	4	0	0

Bxb1.12.79	9	8	6	1	0

Bxb1.12.81	9	7	3	0	0

Bxb1.12.82	9	8	7	0	1

Bxb1.12.83	9	9	3	1	0

Bxb1.28.1	27	26	22	6	0

Bxb1.28.2	18	18	14	0	0

Bxb1.28.3	16	16	11	1	0

Bxb1.28.4	9	8	8	3	0

Bxb1.28.6	26	24	24	11	0

Bxb1.28.7	17	15	13	0	0

Bxb1.28.8	3	3	3	2	0

Bxb1.28.9	18	17	17	4	0

Bxb1.28.10	23	22	16	3	0

Bxb1.28.11	8	7	7	2	0

Bxb1.35.1	18	9	5	0	0

Bxb1.35.2	36	28	17	7	0

Bxb1.35.3	103	47	27	1	9

Bxb1.35.15	23	19	14	5	0

Bxb1.35.29	24	23	23	8	0

Bxb1.35.32	23	23	23	0	0

Bxb1.35.33	23	19	16	1	0

Bxb1.35.36	24	23	23	5	0

Bxb1.35.41	15	14	9	0	0

Bxb1.35.44	17	17	16	0	0

*^a ^*Primers ***e ***and ***f ***yielded the 1.23 and/or 0.43 kb target fragment

*^b ^*Primers ***e ***and ***f ***yielded the 0.43 kb excision fragment

*^c ^*Primers ***g ***and ***h ***failed to detect the 0.72 kb *Bxb1 *fragment

*^d ^*Primers ***g ***and ***h ***yielded the 0.72 kb *Bxb1 *fragment and primers ***e ***and ***f ***failed to detect the 1.23 and/or 0.43 kb target fragment

For the nine TR_1_-Bxb1.9 plants that were backcrossed, 77% of the progeny plants (177 of 229) that harbor the target locus showed excision of the *attP *and *attB*-flanked DNA, with 21% (37 of 177) lacking the recombinase gene (Table 2). Of the TR_1_-Bxb1.12 plants, 61% (43 of 70) of target plants showed excision of the *attP *and *attB*-flanked target, and 9.3% (4 of 43) lack the recombinase gene (Table 2). A total of 87% of the TR_1_-Bxb1.28 plants (135 of 156) harbor the target locus with excision of the *attP *and *attB*-flanked DNA, 24% (32 of 135) lack the recombinase gene (Figure 2c, d; Table 2). Out of the ten TR_1_-Bxb1.35 plants that were backcrossed, 78% of the plants (173 of 222) that harbor the target locus showed excision of the *attP *and *attB*-flanked DNA, with 16% (27 of 173) lacking the recombinase gene. The genomic excision 0.43 kb PCR product from two representative individuals from each family was sequenced and examined for conservative recombination. All eight Bxb1-mediated excision PCR products sequenced were conservative and site specific (Figure 1e).

### Molecular confirmation of the BC_1 _progeny

**Figure 3:**
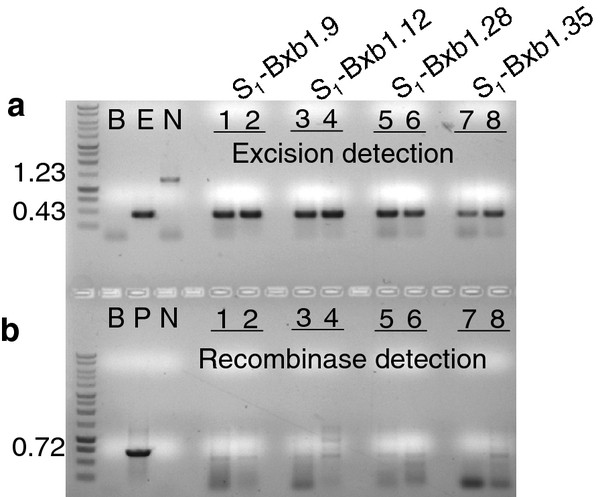
**PCR analysis for site-specific recombination and the presence of the recombinase gene in the selfed (S_1_) generation**. PCR reactions (**a**) with primers ***e ***and ***f ***or (**b**) with primers ***g ***and ***h ***on representative plant DNAs. **a, b**) Selfed lines (lanes 1, 2) S_1_-Bxb1.9; (lanes 3, 4) S_1_-Bxb1.12; (lane 5, 6) S_1_-Bxb1.28; (lane 7, 8) S_1_-Bxb1.35 positive for excised target but the *Bxb1 *transgene is absent. Control lanes are B (blank, no DNA); E (excision, pN6-Bxb1_exc _DNA); N (no excision, pN6-Bxb1); P (pCOXS3-Bxb1).

**Figure 4:**
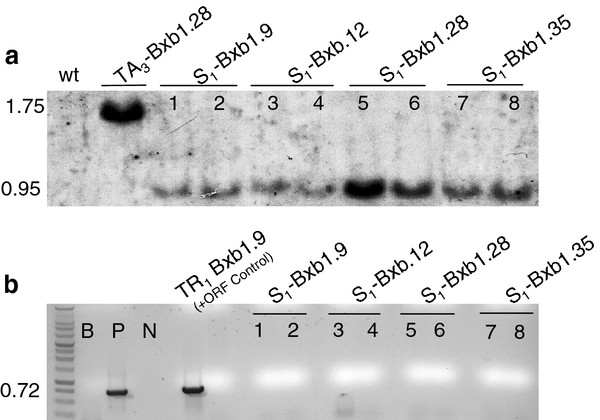
**S_1 _plants examined by Southern blot analysis and whole plant genomic PCR**. (a) Genomic DNA cleaved with *Eco*RV hybridized with a ^32^P-labeled GUS1350 probe (Figure 1). **(b) **Genomic DNA PCR amplified with with primers *e *and *f*. Plant lines (lanes 1, 2) S_1_-Bxb1.9; (lanes 3, 4) S_1_-Bxb1.12; (lane 5, 6) S_1_-Bxb1.28; (lane 7, 8) S_1_-Bxb1.35. Control lanes are wt (wild type *Arabidopsis *DNA) and TA_3_-Bxb1.9, (target lines).

BC_1 _plants that showed excision but lacked the recombinase gene were self-fertilized to generate progeny designated S_1_-Bxb1. PCR analysis on these plants confirmed the inheritance of the excision event in the absence of the *Bxb1 *recombinase gene (Figure 3), which indicates germinal transmission. For further molecular confirmation, Southern blot hybridization was conducted on two selected S_1 _individuals. The genomic DNA was isolated and cleaved with *Eco*RV, which is expected to liberate either a 1.60 kb or a 0.95 kb fragment from the non-recombined or recombined structure, respectively (Figure 1a, c). The GUS1350 probe detected the 1.60 kb band in the parental lines but not in the S_1 _plants (Figure 4a, lanes 1-8). Instead, only the 0.95 kb band was observed for S_1 _plants from the TR_1_-Bxb1 lineage. The genomic DNA was also PCR amplified with primers ***g ***and ***h ***(Figure 1b), which would generate a 0.72 kb fragment if the genome were to harbor a COXS3-Bxb1 T-DNA. Whole plant genomic DNA was PCR amplified to ensure the absence of the *Bxb1 *transgene since previous screening methods only used DNA from individual leaves and could miss mosaic plants. Amplification detected the *Bxb1 *gene fragment in the parental control plants but not in the S_1 _plants, validating that these excision positive plants were recombinase negative (Figure 4b, lanes 1-8).

**Figure 5:**
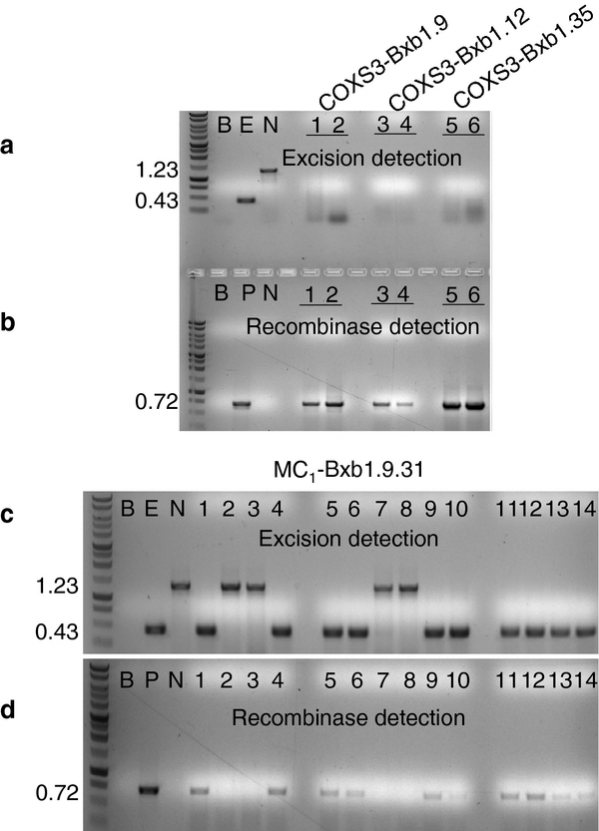
**PCR analysis for site-specific recombination and the presence of the recombinase gene the manual cross (MC_1_) generation**. PCR reactions (**a, c**) with primers ***e ***and ***f ***or (**b, d**) with primers ***g ***and ***h ***on representative plant DNAs. **a, b**) Segregated lines (lanes 1, 2) S_1_-Bxb1.9; (lanes 3, 4) S_1_-Bxb1.12; (lane 5, 6) S_1_-Bxb1.35; (lane 7, 8) S_1_-Bxb1.28 negative for excised target but positive for Bxb1 gene. As target line TA_3_-Bxb1.28 appears to be homozygous no Bxb1 expression lines were derived from the retransformation event. **c, d**) MC_1_-Bxb1.9.31 with predicted, excision events (0.43 kb) only appearing in lanes when the recombinase gene is present (0.72 kb). Control lanes are B (blank, no DNA); E (excision, pN6-Bxb1_exc _DNA); N (no excision, pN6-Bxb1); P (pCOXS3-Bxb1).

We further isolated, by segregation, *Bxb1 *recombinase expression lines for the purpose of crossing to the original TA_3 _target lines to determine if a genomic excision event could be facilitated using this alternative approach. Two independent lines from each of three (TR_1_-Bxb1.9, 12, and 35) secondary transformation events were isolated from segregating populations via PCR (Table 2) and designated lines COXS3-9.17, 9.31; COXS3-12.69, 12.72 and COXS3-35.15, 35.33. Recombinase expression lines were not isolated from the TR_1_-Bxb1.28 transformation event due to the apparent homozygosity of original TA_3_-Bxb1.28 target line (Figure 5a, b; Table 2). Each of these six independently isolated lines was crossed to the original target line TA_3_-Bxb1.28. The manually crossed progenies (MC_1_) were grown without selection and screened by PCR for the target locus (primers ***e ***and ***f***) and the recombinase gene (primers ***g ***and ***h***; Figure 5c, d). Of the MC_1_-Bxb1.9 plants that carried both the target locus and *Bxb1 *gene, 100% (47 of 47) of the tested individuals displayed the 0.43 kb excision band in the absence of the unexcised 1.23 kb target band when screened using PCR (Table 3; Figure 5c, d). Of the MC_1_-Bxb1.12 plants with both the target and *Bxb1*, 80% (17 of 21) generated only the 0.43 kb excision PCR product (Table 3). While 63% (37 of 59) of the MC_1_-Bxb1.35 individuals generated only the 0.43 kb PCR product derived from an excised genomic target (Table 3).

**Table 3:** PCR analysis of MC_1 _plants

MC_1_-Parent line	Plants tested	**Positive for target locus **^***a***^	**Positive for recombinase gene **^***b***^	**Positive for excision and recombinase gene**^***c***^	Positive for **excision and negative for unexcised product **^***d***^
Bxb1.9.17	31	30	29	28	28

Bxb1.9.31	31	31	19	19	19

Bxb1.12.69	16	16	14	14	14

Bxb1.12.72	16	15	7	7	3

Bxb1.35.15	28	26	25	24	24

Bxb1.35.33	46	45	36	35	13

*^a ^*Primers ***e ***and ***f ***yielded the 1.23 and/or 0.43 kb target fragment

*^b ^*Primers ***g ***and ***h ***yielded the 0.72 kb *Bxb1 *fragment

*^c ^*Primers ***e ***and ***f ***yielded the 0.43 kb excision fragment and primers ***g ***and ***h ***detect the 0.72 kb *Bxb1 *fragment

*^d ^*Primers ***e ***and ***f ***yielded the 0.43 kb excision fragment and failed to detect a 1.23 kb target fragment

## Discussion

This research investigated the Bxb1 recombination system as a potential tool for the precise removal of plant transgenes. Use of such technology potentially allows the production of crops with increased yield, resistance to key stresses like disease and drought [17], improve bioenergy production, and new food products that provide valuable health benefits while eliminating the retention of unneeded "marker" genes. This in turn addresses concern relative to both human consumption and potential for transfer of these transgenes that confer antibiotic or herbicide resistance to organisms within the environment [18]. Recombinase-mediated genetic engineering provides a favorable method for enhancing the precision of biotechnology allowing not only marker removal but also facilitating site-specific introduction of a transgene as a single copy into the genome thereby eliminating the random nature of conventional transgene integration. Our interest in site-specific recombination lies in its ability to facilitate crop improvement through controlled engineering of the plant genome. The Bxb1 serine recombinase has uni-directional recombination activity and has the capacity for excision, inversion and integration within the plant genome.

The strategy as initially designed was based on the assumption that we could use *gusA *expression as a reporter of site-specific recombination. The pattern of GUS enzyme activity would reveal genomic excision of the target sequence and any tissue specificity in the observed recombination activity. This strategy, however, failed to perform as desired with initial excised plants having either weak GUS staining or not producing any detectable β-glucuronidase activity. Subsequent analysis of the original TR_1_-Bxb1 progeny confirmed that use of reporter enzyme activity was an unreliable indicator of excision. We had also observed this phenomenon with other constructs used in both *Arabidopsis *and *S. pombe *[5,6,15]. It is possible that the 47 bp *attP/B *hybrid sequence present within the transcript leader sequence of the *gusA *gene may cause poor expression due to methylation or by some other mechanism that inhibits gene expression. Due to this circumstance, the analysis and scoring of site-specific excision were performed using PCR.

Site-specific excision was detected in all TR_1_-Bxb1.9, TR_1_-Bxb1.12, TR_1_-Bxb1.28 and TR_1_-Bxb1.35 plants. The majority (72%) of the plants containing excision of the target sequence were exclusively positive for the 0.43 kb excision band in the absence of the 1.23 kb unexcised target band. This suggests that efficient Bxb1-mediated excision of the four target lines within the *Arabidopsis *genome. Indeed, when segregants derived from TR_1 _lines, containing only the *Bxb1 *expression cassette were manually crossed with TA-Bxb1.28 target plants, 80% (101/127) of the progeny generated only the 0.43 kb excised target PCR product, again independently demonstrating that *Bxb1 *functions well in these plants. From analysis of the BC_1 _plants, 78.0% (528 of 677) of those derived from the four TR_1_-Bxb1 lines showed evidence of excision, while in a previous line of research 77.3%, 85.6% and 99.6% of the BC_1 _plants of the TR_1_-ParA, phiC31 and Cre lines, respectively exhibited excision [6,15]. By this measure, it appears that the native Bxb1 recombinase mediated excision occurs with efficiency comparable to ParA and phiC31 and approaching that of the Cre-*lox *system. It is possible that through optimization and or the addition of a nuclear localization signal that Bxb1 can be enhanced to even higher levels of activity in plants. Although, the majority of the BC_1 _lines displayed excised genomic target, it is difficult to give a precise quantitative assessment of the *Bxb1 *activity since only a modest number of different target locations were thoroughly characterized and direct comparison to other recombinases was not addressed. Variability in copy number and chromosome locations of the *Bxb1 *gene can affect the amount of recombinase protein produced and thus may impact the efficiency of the excision reaction observed, making a direct comparison difficult.

As an alternative method of recombinase introduction into the plant target lines, hand pollination between *Bxb1 *recombinase expressing plants and pN6-Bxb1 target plants was performed. PCR analysis of the manually crossed MC_1 _progeny demonstrated that this is a viable method for the generation of individuals with genomic target excision (Figure 5). However, it was observed that like secondary *Agrobacterium *transformation with the recombinase expression cassette, the genomic excision results varied between lines (Table 3). Use of a demonstrated recombinase expression line such as COXS3-9.17 enabled sufficient recombinase mediated excision events to excise all target DNA when crossed together. It was also observed that segregation of the secondary *Agrobacterium *transformed TR_1 _lines, without benefit of backcrossing, produced excised target and recombinase expression-only T-DNA lines in the TR_2 _and TR_3 _generations (data not shown). This indicates that the *Bxb1 *expression T-DNA in these lines was at a single locus or a low number loci within the genome and that expression was sufficient to facilitate recombination, allowing segregation by self-pollination.

Since PCR assays of genomic DNA from leaf tissue only indicates that excision has occurred in somatic cells, we utilized Southern blot analysis to ascertain whether target sequence removal had occurred in the germline. As long as *Bxb1 *DNA was present in the genome, or the Bxb1 protein was present in the germline cells, the possibility that recombination was generated *de novo *could not be ruled out. Hence, BC_1 _plants were screened by PCR for the absence of the *Bxb1 *recombinase gene, and the following generation (S_1 _plants) was confirmed by Southern blot hybridization. As is shown in (Figure 4 lanes #1 - 8) germinal transmission of the genomic excision event in the absence of the *Bxb1 *recombinase gene occurred, illustrating that the production of stable lines with the unwanted DNA removed can be achieved.

Precise genomic integration is another benefit of recombinase technology and allows the use of more sophisticated recombinase applications [4]. Production of precisely controlled transgenic plants through site-specific integration has been reported to function in *Arabidopsis*, tobacco and rice [19-28] with Cre, Flp and R recombinase systems. The Bxb1 recombinase with its uni-directional catalytic activity presents an alternative way to facilitate stable site-specific integration events without the elaborate strategies required by the bi-directional systems. Peer-reviewed literature reported that Bxb1 is capable of genome targeting in insect cells [13] and targeted integration into the genome of tobacco [7]. Utilization of Bxb1 for genome modification could be facilitated through the identification of cryptic *attP *or *attB *sites as potential locations for transgene introduction as shown by work with phiC31 in the mammalian genome [11].

As our lab is interested in pursuing targeted integration using active 'cryptic' sites, we searched the *Arabidopsis thaliana *genome for the presence of sequences similar to the Bxb1 *att *sites. Using BLASTn and FuzzNuc http://embossgui.sourceforge.net/demo/fuzznuc.html searches we identified 27 *Arabidopsis *sequences with > 60% nucleotide identity with the minimal 48 bp *attP *and 42 bp *attB *sites. A total of eighteen sequences had 29 or 30 matches (60-63% identity) to the 48 bp *attP *sequence, while nine sequences had 27-29 nucleotides in common (64-69% identity) with the 42 bp Bxb1 *attB *sequence (Additional file 1: Figure S1a, b). While nine of these sequences contained the conserved central core "GT" sequence (Figure 1d) required for Bxb1-mediated recombination, most of these sequences did not (Additional file 1: Figure S1a, b). Since the dinucleotide core of the Bxb1 *att *sites is required for both homology matching and site orientation in the recombination reaction [9], we repeated the Bxb1 *att*-like sequence genome search using only the core sequence conserved between the *attP *and *attB *sites as the query (Figure 1d, red text). This second search identified nine sequences which contain only two mismatches compared to the conserved core *att *sequence. Five of these sequences contained 50-56% overall identity with *attP *(Additional file 1: Figure S1c) and six with 50-62% overall identity with *attB *(Additional file 1: Figure S1d). Most of these contained the required GT core sequence. Two of these sequences (marked with an asterisk in (Additional file 1: Figure S1c, d), were among the most similar to both *attP *and *attB *sequences. Next we mapped these 36 *att*-like sequences onto the five *Arabidopsis *chromosomes. A diagram displaying the location and orientation of the *att*-like sequences is shown in (Additional file 2: Figure S2). The closest directly oriented sites are located approximately 105 kb apart near the telomere of chromosome 4. Although both of these sequences have high overall identity with *att *sites (> 62%), neither contains the conserved GT core sequence (Additional file 1: Figure S1, Additional file 2: Figure S2) making Bxb1-mediated recombination highly unlikely. Similarly, there are no instances in the genome where *attP*- and *attB*-like sequences both contain the central dinucleotide "GT" and occur in direct orientation on the same chromosome (Additional file 1: Figure S1, Additional file 2: Figure S2). Taken together, the limited overall sequence similarity to *attP *and *attB*, the lack of the required central "GT" within most of the *att*-like sequences, and their dispersed chromosomal location within the genome, suggest that the potential for Bxb1-mediated recombination of endogenous *Arabidopsis *sequences within the genome is quite low. Consistent with this observation, the *OXS3 *promoter-*Bxb1 *transgenic plants did not exhibit compromised viability, morphological or growth defects like was observed earlier for *35S-phiC31 *transgenic *Arabidopsis *plants which commonly exhibited crinkled leaves [29]. The presence of such phenotypic aberrations clearly indicate the importance of utilizing controlled recombinase transgene expression with the appropriate use of promoters.

## Conclusion

This research demonstrates that the Bxb1 recombinase successfully performs site-specific genome modification in *Arabidopsis *germline tissue. Plants actively expressing the *Bxb1 *transgene appeared phenotypically normal with seed set equivalent to wild type, suggesting that there are no detrimental impacts from constitutive expression. The excision events produced by Bxb1 were conservative site-specific deletions of the *attP *and *attB*-flanked DNA from the plant genome. In a majority (20 out of 39) of the transgenic lines examined, at least one BC_1 _segregant was recovered that contained a germinally transmitted excision event lacking the *Bxb1 *transgene. These results were molecularly validated with DNA blot hybridization and show that the secondary transformation strategy used in this study can be utilized to generate marker gene-free transgenic plants. This type of approach will be particularly useful in plants where cross pollination is either not possible or undesirable. In addition, we show that an alternative approach to marker gene removal using cross pollination between recombinase expressing and *att*-flanked target containing transgenic lines is also a viable strategy. Therefore, taken together the results clearly illustrate that the Bxb1-*att *system performs genomic excision removing unwanted DNA to generate stable recombinase-free *Arabidopsis *transgenic plants.

## Methods

### DNA constructs

pN6-Bxb1 (Figure 1a): An *Asc*I*-attP-*stuffer*-attB-Nhe*I fragment was retrieved from pPB-Bxb1 [5] and inserted into binary vector pCAMBIA-1301 http://www.cambia.org/daisy/cambia in which the *Nco*I site between *35S *and *gusA *had been changed to *Asc*I and *Spe*I. The vector contains *hptII *(hygromycin phosphotransferase gene) for selection in plants. The pN6-Bxb1_exc _control vector used in control "E" lanes (Figure 2a, c; Figure 3a; Figure 5a, c) was generated by removal of the non-coding stuffer region by Bxb1 recombinase-mediated excision in bacteria.

pCOXS3-Bxb1 (Figure 1b): The *Bxb1 *ORF was Phusion (NEB, New England) PCR amplified with a 5' *Asc*I and 3' *Nhe*I sites (underlined) and inserted into pCOXS3-ParA [15] to generate the final construct. Primers used were 5'-AGTCGGCGCGCC ATGAGAGCCCTGGTAGTCATCCG -3' and 5'-AGTCGCTAGC TCAAGACATTCCAGTATGAAGTCTTTCAACAACAGATCC -3'.

The 1.5 kb fragment promoter of the *OXS3 *gene (At5g56550) from *Arabidopsis thaliana *(ecotype: L*er*) was used to express the *Bxb1 *gene, as previously described [15,16]. The pCAMBIA-2300 http://www.cambia.org/daisy/cambia, a binary vector with *nptII *(neomycin phosphotransferase II gene) for plant selection was used as the backbone for plant transformation.

*Agrobacterium tumefaciens *GV3101 was used for transformation of *Arabidopsis *(ecotype: L*er*) by the floral dip method [30] modified by adding 0.01% Silwet L-77 (Lehle Seeds, Round Rock, TX) to the infiltration medium. Primary transformants were selected on MS medium (Sigma), 1% sucrose, 0.7% agar with 20 μg/ml hygromycin or 50 μg/ml kanamycin as needed for 10 days prior to cultivation in soil.

### PCR analysis

Genomic DNA was extracted by grinding a single leaf in 400 μl of buffer (200 mM Tris HCl pH 7.8, 250 mM NaCl, 25 mM EDTA, 0.5% SDS). After centrifugation and isopropanol precipitation, the pellet was washed with 70% ethanol and resuspended in 50 μl of water. PCR amplication was performed using two μl of genomic DNA in reactions with a total volume of 25 μl. Primers were (Figure 1): ***e ***(5'-ATATCTCCACTGACGTAAGG-3'), ***f ***(5'-ATCATCATCATAGACACACG-3' for N6-Bxb1); ***g ***(5'- ATGAGAGCCCTGGTAGTCATCCG-3'), ***h ***(5'-CGGAGATCATCGATCGCTTCAGC-3' for *Bxb1*). Gel images were digitized with a resolution of 200 dpi in black on white background TIF format.

### Southern blot analysis

Genomic DNA was extracted from plant aerial portions using a modified cetyl-trimethyl-ammonium bromide method as described [31]. The 0.79 kb GUS1350 ^32^P-labeled probe was produced by *Taq*™ polymerase (Promega) using primers 5'-CAAGACCCTTCCTCTATATAAG-3' and 5'-CGAGTTCATAGAGATAACCTTC-3'.

## Competing interests

The authors declare that they have no competing interests.

## Authors' contributions

JT designed the approach, constructed the plasmids, collected data and interpreted the research results. JT supervised RC, prepared and submitted the manuscript. RC and JS provided technical assistance with plant maintenance, DNA preparation, PCR data collection and analysis. Participated with manuscript preparation and editing. RT provided bioinformatics research on the cryptic *attB *and *attP *sites and performed Southern blot hybridization and analysis. Participated with manuscript preparation and editing. YY and YW provided assistance on background studies, data interpretation and manuscript editing. DO provided data interpretation and manuscript editing. All authors read and approved the final manuscript.

## Supplementary Material

Additional file 1 **Figure S1 *Arabidopsis *sequences with the highest similarity to Bxb1 *attP *and *attB *sites**. (**a**) Alignment of the 48 bp *attP *site with 18 sequences from the *Arabidopsis *genome that have 60% or greater overall identity is shown. (**b**) Alignment of the 42 bp *attB *site with the nine sequences from the *Arabidopsis *genome that have 64% or greater identity. (**c**) Alignment of the 20 bp conserved Bxb1 core *att *sequence with the five best core matches (2 mismatches to Bxb1 *att *core) with 50% or greater identity with *attP*. (**d**) Alignment of the 20 bp conserved Bxb1 core sequence with the six best core matches (2 mismatches to Bxb1 *att *core) with 50% or greater identity with *attB*. Nucleotides identical to the core *att *site are highlighted in red text, while matches beyond the core are highlighted in blue text, other non-matching sequence is in gray. Click here for file

Additional file 2 **Figure 2 Chromosomal alignment of *Arabidopsis *sequences with the highest similarity to BxbI *attP *and *attB *sites**. The position and orientation of the 36 *att*-like sequences are displayed on a diagram of the five *Arabidopsis *chromosomes. Their orientation is shown with blue (*attP*) and red (*attB*) arrowheads. Click here for file
