# Supplementary material for: The Bxb1 recombination system demonstrates heritable transmission of site-specific excision in Arabidopsis
Source: BMC Biotechnol. 2012 Mar 21;12:9. doi: 10.1186/1472-6750-12-9 (PMC3341217; doi:10.1186/1472-6750-12-9)
Supplement: Additional file 1 — Figure S1 Arabidopsis sequences with the highest similarity to Bxb1 attP and attB sites. (a) Alignment of the 48 bp attP site with 18 sequences from the Arabidopsis genome that have 60% or greater overall identity is shown. (b) Alignment of the 42 bp attB site with the nine sequences from the Arabidopsis genome that have 64% or greater identity. (c) Alignment of the 20 bp conserved Bxb1 core att sequence with the five best core matches (2 mismatches to Bxb1 att core) with 50% or greater identity with attP. (d) Alignment of the 20 bp conserved Bxb1 core sequence with the six best core matches (2 mismatches to Bxb1 att core) with 50% or greater identity with attB. Nucleotides identical to the core att site are highlighted in red text, while matches beyond the core are highlighted in blue text, other non-matching sequence is in gray. [file 1472-6750-12-9-S1.PDF]

a

Description	Sequence	Match	%ID
attP	GGTTTGTCTGGTCAACCACCGCGGTCTCAGTGGTGTACGGTACAAACC		
AtCHR4 4802231	AGTTTGTCTGTTCAGGCAGGGCTGCTACTTCGGTTGACGAACCAAACC	30/48	63%
AtCHR4 18375112	GCTTTGTTCAATCTACCAGCACGGCCTAAAGAGATACCATACAAACA	30/48	63%
AtCHR3 16159943	CACTTGTCTACTAATCTAACACGGTCTCAGAAGGGTTAGGAACAAAGT	30/48	63%
AtCHR2 1272791	GGTTTGTCGACACATCATCCACGGGTAAAGCGGTAGACGATACAATAG	29/48	60%
AtCHR3 1715653	GGTTTGATTCATCAATCATAACCTTTTCAAAGGTTTAGCTACCAATC	29/48	60%
AtCHR2 1486673	TGTTTTTGTGGACAACCAAGGCTCTCTCCATATTGGAGTGGAACCACC	29/48	60%
AtCHR5 19900724	CGTTAATTCGGCGATTGATCGTGTTCTTAGTTGTGAATGGAACAAAGC	29/48	60%
AtCHR3 628137	GGTTGATATCTTCTACGAGCTCGGTTTCGGTGGTCCATGGTAAATTGT	29/48	60%
AtCHR3 2702309	GGAGGAATTGGTTAACTCACGCAATCTGATTGGTTTGCGGTAAAACGC	29/48	60%
AtCHR2 11072278	GGTACAACTGGTCAACAGCCGCCTGCTCAAGGTAGTACTGGTCAGGCA	29/48	60%
AtCHR5 13364127	GGAGTCGCTGAACCTCCACCGCGAGATCTCTCGTGAAGGCACAACCG	29/48	60%
AtCHR4 5244886	GTTTTGTATGCTCAAACAAGTAACTCTTAGTCGTTCACGAAACATCTC	29/48	60%
AtCHR2 9045241	TTTTTGTTTGGTCAAACACCGGTGACCAAGTGTTGTGCATCAATCAGA	29/48	60%
AtCHR1 21599379	GAATCCTCTGCTCAAACGCATCTGTTTCTGTGGAGTACCATTGAAATT	29/48	60%
AtCHR1 17229515	TCTTTTGCTTGTCCTTTACCAAGTTCTCAGTATTGTAGTGTATATATC	29/48	60%
AtCHR1 24091798	AGTTGATCTTGCTCGTTAGGCCGGTTTCAGAGGTGAAAGGTACTAAAC	29/48	60%
AtCHR3 17039694	GCTACTTGTTGTGAACTATCTAGATCACCATGATATACGGTACCAAAC	29/48	60%
AtCHR3 3092838	ATCTCTTGTTATCAGTCAATGCGGAGGCAGTGGTGAAAGGAACAAACG	29/48	60%

b

attB	CCGGCTTGTCGACGACGGCGGTCTCCGTCGTCAGGATCATCC		
AtCHR4 18480432	CCGAACCACCCACGACGGCGTCCTCCCACGTCGGAATCATCC	29/42	69%
AtCHR4 8210178	ACAGCTTGACGAAGAGATTGTTCTCTTTCGTCAAGATCAACT	28/42	67%
AtCHR2 11929137	TCGGTTTGTCAACAACAATGGCCTGAGTGGTCAGTACCATTC	28/42	67%
AtCHR5 18770446	TCAGCTTGTTCCCTTTGACGTTCTTCTTGCTCACGATCATCC	27/42	64%
AtCHR4 15124705	CCAAGGAGTTGACGAAGGAAGTATTCCTCGTCTGCATTATCC	27/42	64%
AtCHR3 10221180	ACAGCACGTGGACGACGGCTGTCGTCGTCCTCCGGCGTATTT	27/42	64%
AtCHR2 3575808	CGGTGGTGTCGAGGATGGTGGTGGCAGTGGTCAAGAGCATGG	27/42	64%
AtCHR1 18307023	TCCCCTTTCTCTCCACCGTCGTCTCCGTCGTTATGATCTTCC	27/42	64%
AtCHR1 2154150	CTGGCTCTTCTGCCACGGTGGTCTCCTTCCTCACCATGGTAA	27/42	64%

c

BXB1 att core	ACNACNGCGGTCTCNGTNGT		
AtCHR4 10943111*	GTTTCTTCTCCTCCACGACCGCGGTTTCAGGGGTCTCGCCTTTCTTTG	27/48	56%
AtCHR5 7197814	GCTACCAGAGGAGCACCACTGCGATCTCAGTCGGTTAGTCTCACATCT	25/48	52%
AtCHR3 23005212*	GCCTGATGAGTTCTACTTCAACGGTCTCCGTCGTGGACTTCTCGCTTC	25/48	52%
AtCHR2 18076683	GCTCTCTTGGAACTACAACTACGGTCTCTGTGGCAAAGCCTTGGACGA	24/48	50%
AtCHR2 3424592	TGGAAACCAGAAGCACGACCGAGGTCTTCGTAGTCTAAAATAATAAAA	24/48	50%
attP	GGTTTGTCTGGTCAACCACCGCGGTCTCAGTGGTGTACGGTACAAACC		

d

BXB1 att core	ACNACNGCGGTCTCNGTNGT		
ATCHR2 13655360	ACCACCGCTTTAAAAAGGCAGTCTCCGTCGTCATCTTCATCG	26/42	62%
ATCHR4 17369861	CCGCCACCGCAACAACCACCGTCTCCGTCATCCACAACTTCC	26/42	62%
AtCHR4 10943111*	TCTTCTCCTCCACGACCGCGGTTTCAGGGGTCTCGCCTTTCT	24/42	57%
AtCHR1 28616523	GCGCTACCCTTACTACAACCGTCTCCGTCGTCTCCTTCCTCT	23/42	55%
AtCHR1 7117922	GCGCAACAATTACTACAACCGTCTCCGTCGTCTTCTTCCTTT	22/42	52%
AtCHR3 23005212*	TGATGAGTTCTACTTCAACGGTCTCCGTCGTGGACTTCTCGC	21/42	50%
attB	CCGGCTTGTCGACGACGGCGGTCTCCGTCGTCAGGATCATCC		

Supplementary Figure 1 ***Arabidopsis*** **sequences with the highest similarity to Bxb1** ***attP*** **and** ***attB*** **sites.** (**a**) Alignment of the 48bp *attP* site with 18 sequences from the *Arabidopsis* genome that have 60% or greater overall identity is shown. (**b**) Alignment of the 42bp *attB* site with the nine sequences from the *Arabidopsis* genome that have 64% or greater identity. (**c**) Alignment of the 20bp conserved Bxb1 core *att* sequence with the five best core matches (2 mismatches to Bxb1 *att* core) with 50% or greater identity with *attP*. (**d**) Alignment of the 20bp conserved Bxb1 core sequence with the six best core matches (2 mismatches to Bxb1 *att* core) with 50% or greater identity with *attB*. Nucleotides identical to the core *att* site are highlighted in red text, while matches beyond the core are highlighted in blue text, other non-matching sequence is in gray.
