# Supplementary material for: The Bxb1 recombination system demonstrates heritable transmission of site-specific excision in Arabidopsis
Source: BMC Biotechnol. 2012 Mar 21;12:9. doi: 10.1186/1472-6750-12-9 (PMC3341217; doi:10.1186/1472-6750-12-9)
Supplement: Additional file 2 — Figure 2 Chromosomal alignment of Arabidopsis sequences with the highest similarity to BxbI attP and attB sites. The position and orientation of the 36 att-like sequences are displayed on a diagram of the five Arabidopsis chromosomes. Their orientation is shown with blue (attP) and red (attB) arrowheads. [file 1472-6750-12-9-S2.PDF]

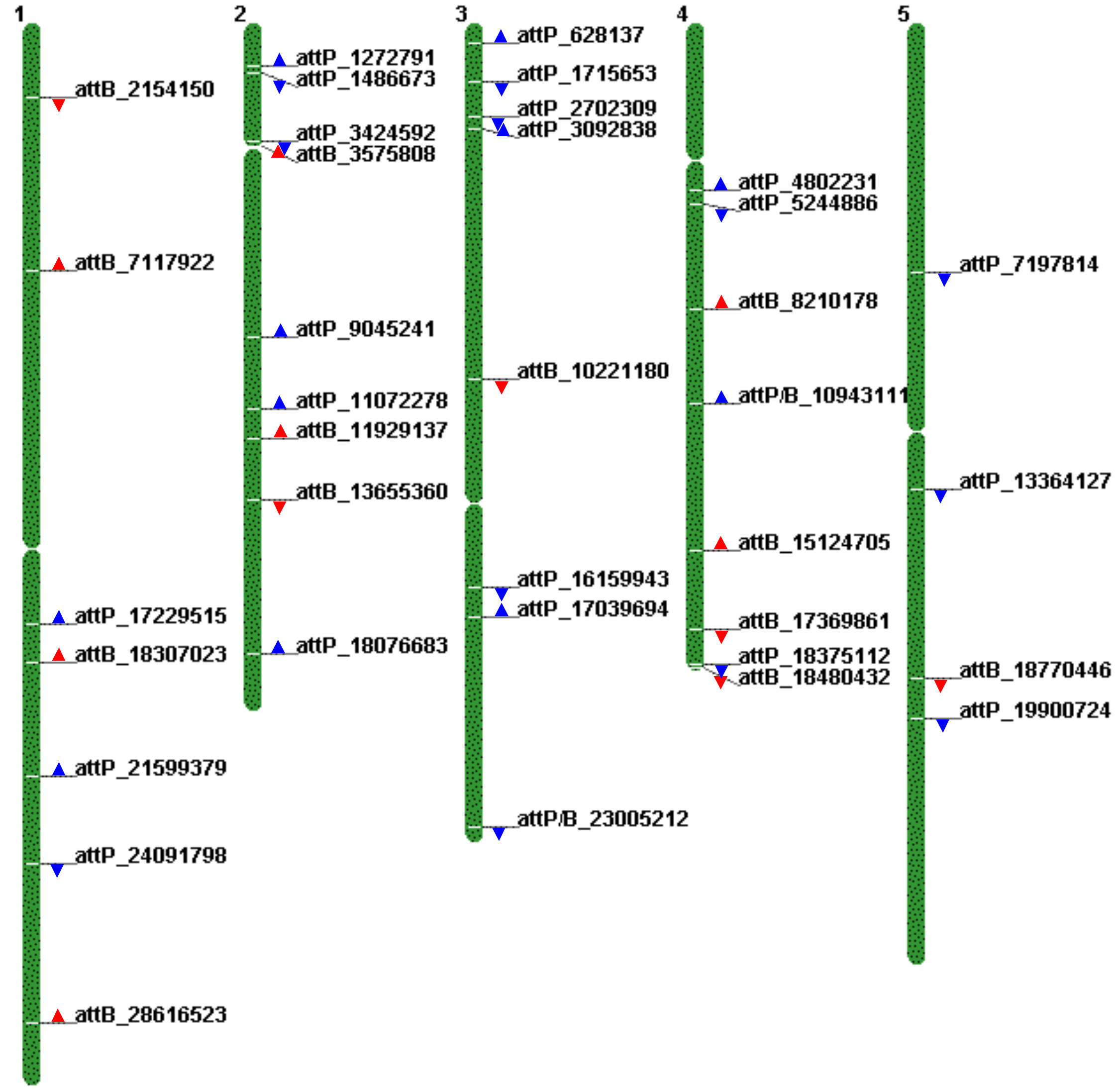

Supplementary Figure 2

Chromosomal alignment of the *Arabidopsis* sequences with similarity to Bxbl *attP* and *attB* sites. The position and orientation of the 36 *att*-like sequences shown in Supplementary Figure 1 are displayed on a diagram of the five *Arabidopsis* chromosomes. Their orientation is shown with blue (*attP*) and red (*attB*) arrowheads.
